# Modeling and forecasting of the under-five mortality rate in Kermanshah province in Iran: a time series analysis

**DOI:** 10.4178/epih/e2015003

**Published:** 2015-01-22

**Authors:** Mehran Rostami, Abdollah Jalilian, Behrooz Hamzeh, Zahra Laghaei

**Affiliations:** 1 Deputy of Public Health, Kermanshah University of Medical Sciences, Kermanshah, Iran; 2 Department of Statistics, Faculty of Sciences, Razi University, Kermanshah, Iran; 3 Research Center for Health Sciences, Department of Health Education and Promotion, School of Public Health, Kermanshah University of Medical Sciences, Kermanshah, Iran

**Keywords:** Fourth Millennium Development Goal, Infant mortality, Time series, Seasonal auto-regressive integrated moving average model, Iran

## Abstract

**OBJECTIVES:**

The target of the Fourth Millennium Development Goal (MDG-4) is to reduce the rate of under-five mortality by two-thirds between 1990 and 2015. Despite substantial progress towards achieving the target of the MDG-4 in Iran at the national level, differences at the sub-national levels should be taken into consideration.

**METHODS:**

The under-five mortality data available from the Deputy of Public Health, Kermanshah University of Medical Sciences, was used in order to perform a time series analysis of the monthly under-five mortality rate (U5MR) from 2005 to 2012 in Kermanshah province in the west of Iran. After primary analysis, a seasonal auto-regressive integrated moving average model was chosen as the best fitting model based on model selection criteria.

**RESULTS:**

The model was assessed and proved to be adequate in describing variations in the data. However, the unexpected presence of a stochastic increasing trend and a seasonal component with a periodicity of six months in the fitted model are very likely to be consequences of poor quality of data collection and reporting systems.

**CONCLUSIONS:**

The present work is the first attempt at time series modeling of the U5MR in Iran, and reveals that improvement of under-five mortality data collection in health facilities and their corresponding systems is a major challenge to fully achieving the MGD-4 in Iran. Studies similar to the present work can enhance the understanding of the invisible patterns in U5MR, monitor progress towards the MGD-4, and predict the impact of future variations on the U5MR.

## INTRODUCTION

The under-five mortality rate (U5MR) is a key pointer of child well-being including health status, and is also a broad indicator of social and economic progress [1,2]. It is one of the main indicators for assessing and monitoring progress in child health status with respect to the United Nations’ Millennium Development Goal 4 [1-4]. The target of the Fourth Millennium Development Goal (MDG-4) is to reduce the U5MR by two-thirds between 1990 and 2015 [2,5]. The U5MR is defined as the probability (per 1,000 live births) that a child will die before reaching the age of five if subject to current age-specific mortality rates [2]. Despite global population growth in recent decades, substantial progress has been made towards achieving MDG-4 in most countries of the world [1,2,6], as well as in Iran [6-9]. The number of under-five deaths worldwide has declined from nearly 12.7 million in 1990 to 6.3 million in 2013 [2]. Consequently, the global U5MR has dropped from almost 90 deaths per 1,000 live births in 1990 to 46 in 2013 [2]. However, this progress is not equally distributed at national and sub-national levels [2,4,10].

Iran is a country that has experienced considerable reductions in the U5MR over the past decades [11]. In Iran, the U5MR was estimated as 281 per 1,000 live births in 1970 to 1975 [6] and declined from 46 per 1,000 live births in 1993 to 25 in 2005 [2]. Despite these gains, it has been seen that the decline in the U5MR across the country is heterogeneous and unequally distributed among provinces [2,12,13]. For example, in 2004, the estimate of the U5MR using data from the death registration system of the Ministry of Health and Medical Education was 28 per 1,000 live births for the whole country, while it was 32 per 1,000 live births in Kermanshah province in the west of Iran [11]. Thus, assessing fluctuations in the U5MR at the national level might not be sufficient and sub-national studies can provide a more detailed understanding of heterogeneity in the U5MR across the country. Moreover, since the U5MR is not constant over time, it is also important to understand how the rate evolves over time and explain its stochastic variations using a valid statistical model.

The main purpose of this study was to detect any statistically significant features in the monthly U5MR in Kermanshah province from 2005 to 2012 and find an appropriate time series model that could adequately explain variations in the monthly U5MR. The study period of 2005 to 2012 was chosen because the child mortality data were available to the authors only for these years at the time of conducting the study.

## MATERIALS AND METHODS

### Socio-demographic characteristics

Kermanshah province is an undeveloped province located in the west of Iran and comprising 14 counties. Based on the population and housing census data of 2011, the population of Kermanshah province was 1,945,227 residents (about 2.6% of the Iranian population, 78 persons/km^2^ and 69.7% urbanization rate) with mainly Kurdish ethnic background [14].

### Ethics statement

This study is based on the digital file of under-five mortality records available from the Deputy of Public Health, Kermanshah University of Medical Sciences. The data were anonymized and deidentified before usage and hence no informed consent was required for this work.

### Data source

The monthly frequencies of under-five mortality in Kermanshah province were extracted from the digital file of the province mortality records from 2005 to 2012 (based on Iranian calendar time) in the provincial health jurisdiction. In this mortality digital file, under-five death records were collected from hospitals, health centers and health houses located in the province. There are few studies on sub-national child mortality in Iran, but a published report by the Iranian Ministry of Health showed that in 2010, more than 91% of mortality in the neonatal period and more than 63% of mortality 1 to 59 months after birth occurred in Iranian hospitals [15]. The frequencies were converted to rates per 1,000 live births using the number of under-five mortalities in each month as the numerator and births in the same month in the province as the denominator. Real times of live births were extracted from the National Organization for Civil Registration. A sequence of 96 monthly U5MRs was obtained and studied for temporal variations. Through the following steps, a time series analysis has been conducted on the data in order to identify structural patterns in the monthly U5MR in Kermanshah province from 2005 to 2012 and a short-term (six months) prediction has been made. See Appendix 1 and references therein for the technical details of the time series analysis.

### Data preparation

**Figure 1.**
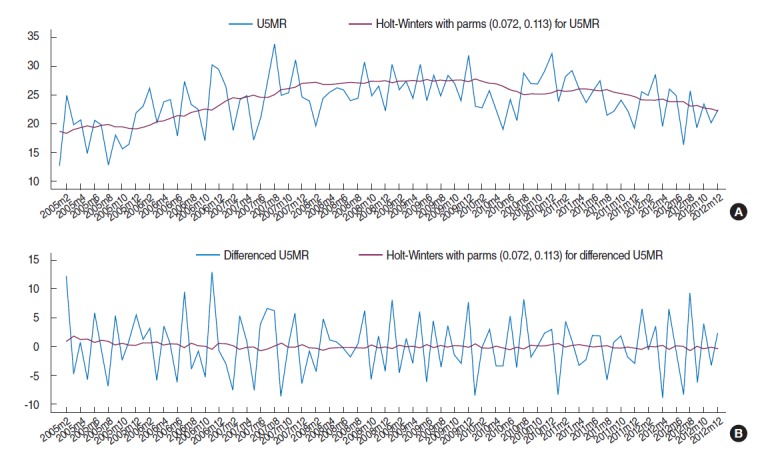
Time series plot of (A) the original and smoothed under-five mortality rate (U5MR) data and (B) differenced U5MR.

Box-Cox transformation for assessment of stability in the data variance (p=0.20 for the null hypothesis of θ=1) shows that variance of the U5MR series is constant over time and no variance-stabilizing transformation is required. The Holt–Winters smoothing was applied to the U5MR series to examine any trends in the data. The time series plot of the original and the smoothed U5MR data are shown in Figure 1A. No seasonal or periodic components are clearly apparent in this plot but the smoothed series suggests an increasing trend, especially in the early years of the study period. Prais-Winsten regression also confirms (t=2.20, p=0.030) the presence of a significant linear trend with a positive slope. The augmented Dickey-Fuller unit root test accepts (lags=3, Z(t)=-2.51 and approximate p=0.112) the null hypothesis that the U5MR series has a unit root and indicates that the increasing trend in the data is stochastic and is caused by the effect of random shocks. Therefore, successive differencing could be performed on the U5MR to eliminate the unit root and hence the stochastic increasing trend and obtain a zero mean stationary time series. The first difference of the U5MR and its smoothed series are shown in Figure 1B. No trend is recognizable in the smoothed differenced series and the augmented Dickey-Fuller unit root test for the differenced series rejects (lags=3, Z(t) =-6.801 and approximate p<0.001) the null hypothesis of unit root and confirms that the first difference of the U5MR is a stationary time series.

### Model identification and estimation

**Figure 2.**
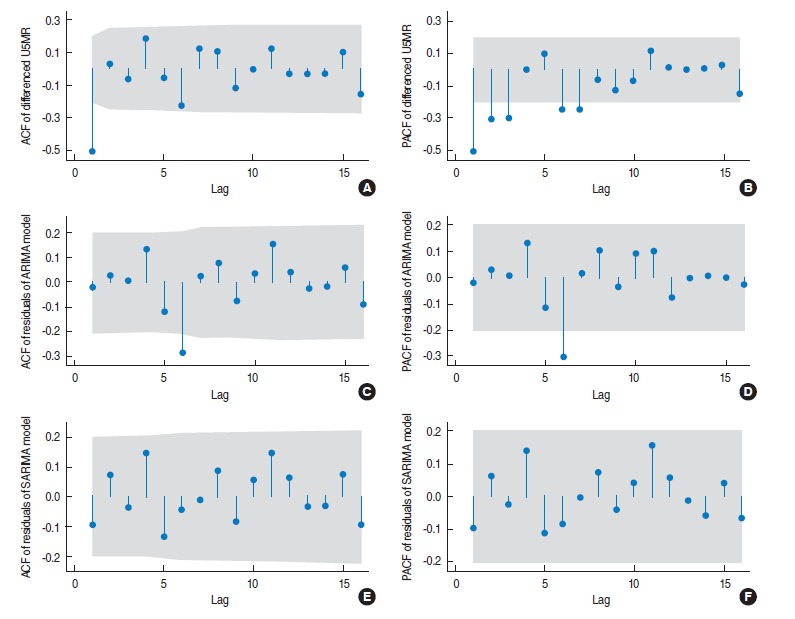
ACF (A, C, E) and PACF (B, D, F) plots of the differenced U5MR series (A, B), the residual of the fitted ARIMA (0, 1, 1) model (C, D), and the residuals of the fitted SARIMA (0,1,1) (0,0,1)_6_ model (E, F). U5MR, under-five mortality rate; ACF, autocorrelation function; PACF, partial autocorrelation function; ARIMA, auto-regressive integrated moving average; SARIMA, seasonal auto-regressive integrated moving average.

The portmanteau Q-test (Q=91.89, p<0.001) and the Bartlett’s periodogram-based test (B=2.46, p<0.001) for white noise reject the null hypothesis of no serial correlation among observations of the differenced U5MR. To check the structure of such correlations, the sample autocorrelation function (ACF) and partial autocorrelation function (PACF) plots of the differenced U5MR series are shown in (A) and (B) of Figure 2. According to the plots, only the first lag of the ACF is significant (lying outside the grey 95% confidence bands) and the first few lags of PACF are decaying. The ACF and PACF of a first order moving average (MA), or auto-regressive integrated moving average (ARIMA) (0, 0, 1) time series have the same pattern and hence an ARIMA (0, 1, 1) model is appropriate for the original U5MR series. After fitting the ARIMA (0, 1, 1) model (Akaike information criterion [AIC]=534.2, Bayesian information criterion [BIC]=541.9) to the U5MR series and obtaining the model residuals, the ACF and PACF of the model residuals are plotted in Figure 2C and 2D. Both the ACF and PACF are significant at lag 6 (local spike) indicating that there is a six-monthly serial correlation in the data that the fitted ARIMA (0, 1, 1) model is not able to explain. This could be caused by a periodic (seasonal) component with period s=6. Thus, several seasonal auto-regressive integrated moving average (SARIMA) (*p, d, q*) (*P, D, Q*)_6_ models with different combinations of SARIMA (*p, d, q*) and (*P, D, Q*) orders were fitted to the U5MR series. Based on the corresponding AIC and BIC values of the fitted models, the best-fitting model was SARIMA (0, 1, 1) (0, 0, 1)_6_ with the smallest value of AIC=526.2 and BIC=533.9.

### Diagnostic checking

**Figure 3.**
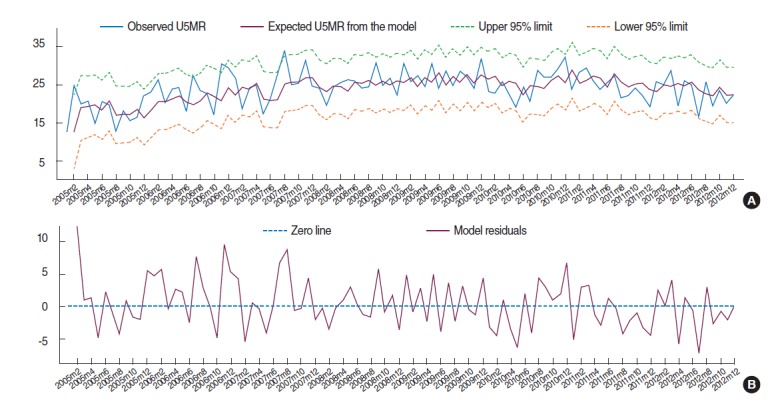
The observed and expected under-five mortality rate (U5MR) from the fitted model with their corresponding (A) 95% confidence limits and (B) model residuals.

According to Figure 3, there is no major discrepancy between the observed and expected U5MR from the fitted model (Figure 3A) and the model residuals vary randomly around zero (Figure 3B). In order to examine the adequacy of the fitted model, the ACF and PACF of the model residuals are shown in Figure (E) and (F) of Figure 2. All lags of ACF and PACF are within the 95% confidence bands, indicating that there is no correlation structure in the model residuals and hence that the fitted SARIMA model is adequate. This is also confirmed by the portmanteau Q-test (Q=37.4, p=0.59) and Bartlett’s periodogram-based test (B=0.60, p=0.86) for white noise, which accept the null hypothesis of no serial correlation in the model residuals. Moreover, the Shapiro-Wilk test (W=0.984, p=0.29), and Skewness-Kurtosis test (χ^2^=3.64, p=0.16) accept the normality assumption of the model residuals. Finally, the ARCH-LM test for heteroscedasticity in variance of the model residuals accepts (χ^2^= 0.004, p=0.951) the null hypothesis of no auto-regressive conditional heteroskedasticity effect. Therefore the overall goodness-of-fit of the model was evaluated.

All analyses were performed using STATA/SE version 12.0 (StataCorp, College Station, TX, USA) [16] and the significance level was chosen at p-value<0.05.

## RESULTS

According to the best-fitting model, the U5MR at month *t*, *x_t_*, is determined by

$$x_{t}=x_{t-1}+z_{t}-0.78z_{t-1}-0.31z_{t-6}+0.24z_{t-7}$$

where *z_t_* is a white noise process with standard deviation ${\hat{\sigma }}_{z}\;$= 3.71. This means that the U5MR of each month is directly influenced by the U5MR of the first preceding month and independent random shocks of the current, first, sixth and seventh preceding months, with their corresponding coefficients. A detailed description of the estimated model parameters is presented in Table 1. It can be seen that both the regular first-order MA parameter and the seasonal MA parameter are statistically significant (p<0.05). Therefore, the fitted model suggests three components: a first-order difference for removing a stochastic increasing trend, a regular MA (1) term for serial correlation in the data and a seasonal MA (1) for reoccurrence of six-monthly dependence structures in the data. A significant regular MA (1) component is common in mortality rate time series and indicates that the random fluctuation in the U5MR of each month contributes to the U5MR of the next month. Thus, the presence of this term in the fitted model determines structured uncertainty in the data and provides better prediction. This component of the model is the most persistent and reliable part of the model and we expect that it remains the same over time. The significant increasing trend of the series over the study period contradicts the national and global decreasing trend. Since the trend is not deterministic, it is likely to be a consequence of poor data collection in the early years of the study period. The unexpected presence of a seasonal term with a periodicity of six months in the fitted model reveals another issue in the data collection method. Since monthly U5MR data of the province must be reported to the Ministry of Health and Medical Education twice every year (in February and August), it is very likely that unclassified and late reports from different months for some public health facilities are accumulated and mixed up in the final reports. Thus, perhaps like the stochastic trend, the seasonal component of the model is caused by poor quality of data and may vanish over time if more improvement in the data quality were to take place.

The six months ahead (short-term) predictions of the U5MR and their corresponding 95% prediction intervals based on the fitted SARIMA model are reported in Table 2. In general, the prediction intervals are wider for time series with a stochastic trend [17] and here relatively wide ranges of prediction intervals indicate the high uncertainty associated with the predictions. Using the most recent and more accurate data, such predictions can be used for monitoring and forecasting progress towards MDG-4.

## DISCUSSION

In this paper, time series analysis of monthly U5MR data in Kermanshah province in the west of Iran was conducted. According to our findings, the U5MR in Kermanshah province during the study period had a stochastic increasing trend. After primary analysis and applying necessary data adjustments, a SARIMA (0, 1, 1) (0, 0, 1)_6_ was selected as the best fitting model and model assessment and goodness-of-fit tests showed that the model can adequately explain the fluctuations in U5MR. Finally, predictions six months ahead of December 2012 and their corresponding prediction intervals were obtained based on the fitted model.

**Figure 4.**
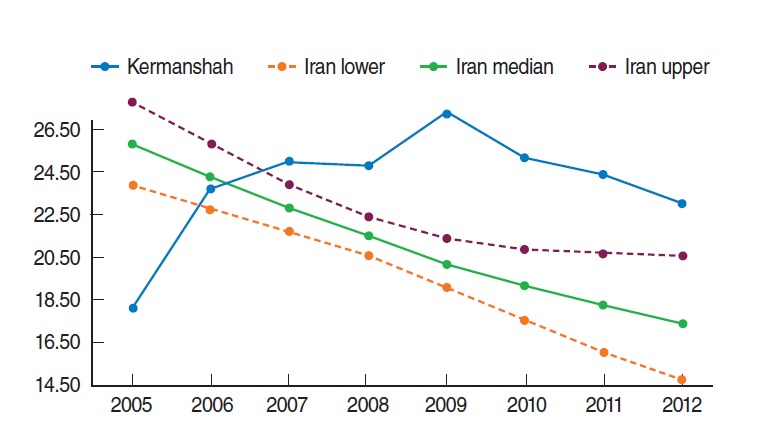
Yearly under-five mortality rate (deaths per 1,000 live births) in Kermanshah province from 2005 to 2012 (resulting from this study) and lower, middle and upper estimates of the yearly under-five mortality rate for Iran. Modified from UN Inter-agency Group for Child Mortality Estimation. Iran (Islamic Republic of): under-five mortality rate [22].

Mortality data sources in developing countries, such as Iran [15,18,19], often suffer from various data availability and data quality issues [1,3,4] and among these issues the under-reporting of deaths and misreporting of ages are common [2,18]. For example, despite considerable efforts to decrease the U5MR in the whole country [2,6-9,11,15], the monthly U5MR in Kermanshah province shows a significant increasing trend during the study period. As demonstrated in Figure 4, the yearly U5MR in Kermanshah province was clearly lower than the estimated national average in 2005 and then higher than the estimated national average from 2007 to 2012. This discrepancy is most likely caused by under-reporting and misreporting of data in the earlier years and improving quality in data collection and registration coverage of U5MR data in the last years of the study period, which is remarkable for the health care system of Kermanshah province. Although there are some demographic methods for assessing causes and estimation of death under-numeration, these methods assume balanced population growth [15]. Iran has undergone drastic demographic changes during the recent three decades. Population growth had a high rate 30 years ago and afterwards dropped to low values [20,21]. These changes make use of demographic methods for estimation of death under-numeration difficult [15]; thus authors were obliged to use the available data for this study.

Besides the possibility that the U5MR has been under-reported, another limitation of this study is that the available data cover a rather narrow time period of eight years due to restrictions in data availability. The third limitation of the study is that, although the model adequacy was evaluated by various statistical methods, the capability of the model for forecasting in short time periods and the accuracy of the predicted values by model are not assessed. The uncertainty of the predictions is assessed only by prediction intervals. However, it should be noticed that generating accurate estimates and predictions of child mortality is a considerable challenge for developing countries [1,2,4,10].

Despite its limitations, the greatest strength of the present study is that it is the first study in Iran that uses time series analysis in order to mathematically model and predict an out-of-sample U5MR. However, additional data from other parts of the country are needed to generalize the results to the national level. In 2015, the MDG-4 target for the Iranian U5MR is 19 deaths per 1,000 live births [2]. It is currently around one year before the 2015 deadline of the goal and substantial progress has been made, but the progress remains insufficient to achieve MDG-4, particularly in Kermanshah province. It can be seen from Figure 4 that despite considerable progress in the past two decades towards reducing the U5MR in Iran [2,10,11], as well as in Kermanshah province [11,15], the burden of child deaths is higher than the nationally estimated and expected levels in this province.

To achieve the MDG-4 on time, reducing under-five mortality inequities among Iranian provinces is an important priority. It seems that one of the major challenges ahead in fully achieving the MDG-4 in Iran is qualitative and quantitative improvement of under-five mortality data collection in health facilities and their corresponding systems. In general, for generating reliable mortality statistics at the sub-national levels it is necessary to improve the reporting and registering of child deaths by health facilities and to make sure that all child deaths that occur in health facilities are of critical importance. This is also crucial in understanding and measuring the impact and effectiveness of plans and interventions in this field. Since the definition of the U5MR indicator has not changed during the recent years, analyzing the invisible patterns and statistical modeling of this indicator within recent years is valuable and may enable health policy-makers to monitor the progress of Iranian child health status in order to evaluate the efficacy of health care systems. Such findings may be useful as they enhance the understanding of the current underlying patterns in the U5MR and monitoring of progress towards the MDG-4. Moreover, using studies similar to the present work, it is possible to predict the impact of future changes on this important child health indicator. Surely, with the expansion of study time periods and improvement of data collection methods, more accurate results will be obtained in the future.

## Figures and Tables

**Table 1.** Detailed description of estimated parameters of the fitted seasonal auto-regressive integrated moving average (0, 1, 1) (0, 0, 1)_6_ model to Kermanshah province monthly under-five mortality rate data

Model parameters	Log likelihood=-260.11		Wald χ^2^=139.91		p<0.001	
	Estimate	Standard error	Z-statistic	p-value	95% confidence interval	
Regular MA (1)	-0.779	0.0719	-10.83	<0.001	-0.92	-0.638
Seasonal MA (1)	-0.306	0.1079	-2.84	0.005	-0.518	-0.095
σ_z_	3.707	0.2919	12.7	<0.001	3.135	4.279

MA, moving average.

**Table 2.** The six-month-ahead predicted values of the under-five mortality rate with corresponding 95% lower and upper prediction intervals based on the fitted seasonal auto-regressive integrated moving average (0, 1, 1) (0, 0, 1)_6_ mode

Time	Predicted value	95% prediction intervals	
		Lower prediction limit	Upper prediction limit
2013m1	24.68	17.4	31.96
2013m2	22.05	14.60	29.49
2013m3	23.69	16.08	31.29
2013m4	23.35	15.59	31.12
2013m5	23.89	15.97	31.81
2013m6	23.43	15.35	31.50

## Appendix 1. TIME SERIES ANALYSIS

### Time series data

A sequence of observations collected at successive points in time, denoted by {*x_t_*}$\overset{n}{t=1}$, is called a time series. Typically, the consecutive observations of a time series are serially correlated. A stationary time series has no deterministic or stochastic trend and/or seasonality, its variance is constant over time and it does not contain any unit root [17,23]. Thus, stationary series fluctuate, with constant variance and no periodic cycles, around a constant mean level and do not systematically increase or decrease over time. In general, modeling and forecasting of stationary time series are greatly simpler than nonstationary time series [17,24]. Thus, as a first step, it is necessary to assess the stationarity of the time series and recognize the potential causes of nonstationarity.

### Data preparation

Smoothing techniques are usually used to reduce irregular roughness (random fluctuations) and detect any persistent pattern such as long term trends or cycles in the data [17,23]. The most common types of smoothing techniques are moving averages (MA), single and double exponential smoothing and Holt-Winters exponential smoothing [16,25]. The Holt-Winters method assumes a linear trend with an intercept and slope that vary over time and filters out the irregular fluctuations using smoothing parameters (weights) [22,24]. The presence of a linear trend with a non-zero slope in the data can be tested using the Prais-Winsten regression method, which uses the generalized least-squares method to estimate the intercept and slope in a linear regression model by allowing errors to be serially correlated [16]. The trend can be deterministic or stochastic [17]. A time series with a deterministic trend reverts to the trend line in the long run and random shocks (fluctuations) have transitory effects on the level of the series [25]. A time series with a stochastic trend or a unit root is not trend reverting and random shocks have permanent effects on the level of the series [23,24]. The hypothesis that the time series contains a unit root (stochastic trend) can be tested using unit root tests like the Augmented Dickey-Fuller test [22,24]. In the case that the presence of a unit root is confirmed, successive differencing can be performed on the data to obtain a zero mean stationary time series. The first lag difference of data, *y_t_=Δx_t_=x_t_-x_t-1_*, can eliminate the unit root [17]. In addition, by subtracting the value of an earlier data from the value of a later data, the first lag differencing can also remove a linear deterministic trend in the data [23,25]. For more complex situations, higher orders of lag differencing might be needed to eliminate several unit roots and/or non-linear deterministic trends [17]. Besides trend, nonconstant variance can also cause nonstationarity [17]. Box-Cox transformation with parameter θ is defined as (*x_t_*^θ^-1)/θ and can be applied to time series in which the variance changes over time in order to stabilize the variance [17].

### Model identification and estimation

The autoregressive integrated moving average (ARIMA) model by Box-Jenkins is the most well-known model for a wide variety of time series [25]. An ARIMA (*p, d, q*) model does not require the original time series *x_t_* to be stationary but it assumes that after d times differencing, the new series *y_t_=Δ^d^x_t_* becomes a stationary zero mean time series. It also assumes that the current value of the differenced series *y_t_* is a linear combination of *p* previous values *y_t-1_, y_t-2_,…,y_t-p_* and *q*+1 values *z_t_, z_t-1_, …, z_t-q_* of a white noise process (independent and identically distributed random shocks with zero mean and common variance ${\sigma_{z}}^{2}$); that is

$$y_{t}=\varphi_{1}y_{t-1}\;+\;\varphi_{2}y_{t-2}\;+\;\cdot \cdot \cdot \;+\;\varphi_{p}y_{t-p}\;+\;z_{t\;}+\;\theta_{1}\;z_{t-1}\;+\;\cdot \cdot \cdot \;+\;\theta_{q}\;z_{t-q}.$$

The autoregressive order *p* is the number of terms in the model that describe the direct dependency among successive observations and the MA order *q* is the number of persistent random shocks that describe indirect dependency among successive observations [25]. They are identified by examining patterns in plots of the autocorrelation function (ACF) and partial autocorrelation function (PACF) of the differenced series *y_t_* [23,25]. After identifying *p* and *q*, the model parameters *φ_1_,…, φ_p_, θ_1_,…, θ_q_* and ${\sigma_{z}}^{2}$are estimated using the maximum likelihood estimation method [17]. If several values for *p* and *q* seem plausible, model selection criteria such as the Akaike information criterion (AIC) and the Schwarz’s Bayesian information criterion (BIC) can be used to select the best fitting model [24].

Seasonal or periodic cycles produce local spikes at specific lags in plots of ACF and PACF [23,25]. Any ARIMA model can be extended to include seasonality and is then called a seasonal autoregressive integrated moving average (SARIMA) model. A SARIMA (*p, d, q*) (*P, D, Q*)_s_ model consists of two multiplicative components: a regular ARIMA (*p, d, q*) component and a seasonal component with autoregressive order *P*, MA order *Q* and difference order *D* [17,26,27]. The seasonal component is responsible for the recurrence of a recognizable pattern after the seasonal periods. The general model equation of a SARIMA (*p, d, q*) (*P, D, Q*)_s_ model has *p* regular autoregressive parameters, *q* regular MA parameters, *P* seasonal autoregressive parameters and *Q* seasonal MA parameters [17]. Similar to the regular ARIMA model, ACF and PACF plots and AIC and BIC criteria can be used to identify the seasonal orders (*P, D, Q*), though in this case the situation is more complicated [17]. Using the maximum likelihood method, all *p*+*q*+*P*+*Q*+1 regular and seasonal parameters of the model can be estimated [23,24].

### Diagnostic checking

After fitting the desired SARIMA model to the observed time series, the model residuals are obtained by subtracting the expected values of observations under the model, ${\hat{\chi }}_{t}\;$, from the true observed values, *x_t_*; that is $r_{t}=\chi_{t}-{\hat{\chi }}_{t}$, [25]. The model residuals contain any features in the data that are not explained by the fitted model. Thus, if the fitted model adequately explains all statistically significant features of the data, then the model residuals must behave as a white noise process [23]. In other words, when the fitted model is good enough for the data, then the model residuals are expected to have no significant features, including trends, seasonality, variance heteroscedasticity and serial correlation. Furthermore, the maximum likelihood estimation method is based on the assumption of normally distributed random shocks *z_t_* which implies that the model residuals be normally distributed [17]. Plots of ACF and PACF of the model residuals are useful in assessing no significant serial correlation in the model residuals. If the fitted model is adequate, both the ACF and PACF of residuals should have no structure to identify [24,25]. In addition, the portmanteau Q-test (goodness-of-fit statistic) and Bartlett’s periodogram-based test for white noise can be also used to test the existence of serial correlation in the model residuals [23]. The adequacy of the normality assumption of the model residuals can be checked using normality tests such as the Shapiro-Wilk, and Skewness-Kurtosis tests [25,28]. Finally, the heteroscedasticity (deviation from constant variance assumption) in the model residuals can be examined by performing an ARCH-LM test for autoregressive conditional heteroscedasticity effects [23,24].

### Prediction and prediction intervals

Once a suitable model has been fitted to the data, the known structure of the model can be used to predict future values of the time series based on previously observed values with a recursive method [23]. Prediction error, the difference between the actual and the predicted values, is inevitable and hence the uncertainty of the predictions must be considered; the farther the prediction beyond the observed values, the less reliable the prediction. For this reason, an interval that with a certain probability (for example 95%) will contain the actual future value, called the prediction interval (PI), is also reported for each prediction [25]. The PI is always wider than the corresponding confidence interval because of the added uncertainty involved in predicting a random variable versus its mean [23,24].
